# Podoplanin-Rich Stromal Networks Induce Dendritic Cell Motility via Activation of the C-type Lectin Receptor CLEC-2

**DOI:** 10.1016/j.immuni.2012.05.022

**Published:** 2012-08-24

**Authors:** Sophie E. Acton, Jillian L. Astarita, Deepali Malhotra, Veronika Lukacs-Kornek, Bettina Franz, Paul R. Hess, Zoltan Jakus, Michael Kuligowski, Anne L. Fletcher, Kutlu G. Elpek, Angelique Bellemare-Pelletier, Lindsay Sceats, Erika D. Reynoso, Santiago F. Gonzalez, Daniel B. Graham, Jonathan Chang, Anneli Peters, Matthew Woodruff, Young-A. Kim, Wojciech Swat, Takashi Morita, Vijay Kuchroo, Michael C. Carroll, Mark L. Kahn, Kai W. Wucherpfennig, Shannon J. Turley

**Affiliations:** 1 Department of Cancer Immunology and AIDS, Dana-Farber Cancer Institute, Boston, MA 02215, USA; 2 Department of Cell and Developmental Biology, University College London, London WC1E 6BT, UK; 3 Division of Medical Sciences, Harvard Medical School, Boston, MA 02115, USA; 4 Department of Medicine and Cardiovascular Institute, University of Pennsylvania, Philadelphia, PA 19104, USA; 5 Immune Disease Institute and Program in Cellular and Molecular Medicine, Children’s Hospital, Departments of Pediatrics and Pathology, Harvard Medical School, Boston, MA 02115, USA; 6 Department of Pathology and Immunology, Washington University School of Medicine, St. Louis, MO 63110, USA; 7 Center for Neurologic Diseases, Brigham and Women’s Hospital and Harvard Medical School, Boston, MA 02115, USA; 8 Department of Biochemistry, Meiji Pharmaceutical University, Kiyose, Tokyo 204-8588, Japan; 9 Department of Microbiology and Immunobiology, Harvard Medical School, Boston, MA 02115, USA

## Abstract

To initiate adaptive immunity, dendritic cells (DCs) move from parenchymal tissues to lymphoid organs by migrating along stromal scaffolds that display the glycoprotein podoplanin (PDPN). PDPN is expressed by lymphatic endothelial and fibroblastic reticular cells and promotes blood-lymph separation during development by activating the C-type lectin receptor, CLEC-2, on platelets. Here, we describe a role for CLEC-2 in the morphodynamic behavior and motility of DCs. CLEC-2 deficiency in DCs impaired their entry into lymphatics and trafficking to and within lymph nodes, thereby reducing T cell priming. CLEC-2 engagement of PDPN was necessary for DCs to spread and migrate along stromal surfaces and sufficient to induce membrane protrusions. CLEC-2 activation triggered cell spreading via downregulation of RhoA activity and myosin light-chain phosphorylation and triggered F-actin-rich protrusions via Vav signaling and Rac1 activation. Thus, activation of CLEC-2 by PDPN rearranges the actin cytoskeleton in DCs to promote efficient motility along stromal surfaces.

## Highlights

► The CLEC-2-PDPN interaction promotes DC migration to LNs ► CLEC-2 activation induces actin-rich protrusions and motility in DCs ► CLEC-2 activation reduces actomyosin contraction and promotes actin polymerization

## Introduction

Cell motility is crucial for trafficking of leukocytes between tissues and for their interaction with one another and their microenvironment. As the quintessential antigen-presenting cell, dendritic cells (DCs) rely on migration via stromal networks to carry antigens long distances from parenchymal tissues to lymph nodes (LNs), where they initiate adaptive immunity and tolerance (Turley et al., 2010). DCs generally reside in parenchymal tissues as immature antigen-capturing cells. Upon encountering pathogens or tissue damage, DCs undergo maturation, which promotes the processing and presentation of major histocompatibility complex (MHC)-peptide complexes, upregulation of costimulatory molecules, and DC migration to LNs (Randolph et al., 2005). Migratory DCs utilize the lymphatics as highways to move to regional LNs where they have the highest chance of interacting with the appropriate antigen-specific T cell.

To reach LNs, tissue-resident DCs must first crawl to and enter afferent lymphatics, which begin as blind-ended vessels within most parenchymal tissues and convey lymph toward the thoracic duct (Randolph et al., 2005). Upon arrival at LNs, DCs traverse the floor of the lymph-draining subcapsular sinus, penetrate the parenchyma, and then crawl along fibroblastic reticular cells (FRCs) in the T cell-rich paracortex. To date, the only well-established directional cues guiding tissue-derived DCs to the LN paracortex are the chemokines CCL19 and CCL21 (Förster et al., 1999; Schumann et al., 2010), two CCR7 ligands expressed by lymphatic endothelial cells (LECs) and FRCs (Link et al., 2007). However, how these chemokines act as functional gradients across such vast distances has not been adequately explained, which raises the question of whether additional hitherto-undiscovered migration mechanisms may also be involved. Recent studies demonstrated that tissue-derived DCs can migrate from tissues to LNs using amoeboid, integrin-independent mechanisms (Lämmermann et al., 2008; Frittoli et al., 2011). However, the specific molecules that support amoeboid movement and initiate cytoskeletal rearrangements to cause spreading and translocation of DCs along stromal scaffolds remain elusive.

We identified the transmembrane glycoprotein podoplanin (PDPN, gp38, T1α) as a candidate molecule that migratory DCs encounter en route to LNs because of its high expression by both LECs and FRCs (Link et al., 2007; Peduto et al., 2009). Interestingly, a steady-state function of PDPN in mature lymphatic vessels and on FRCs has not been defined. Previous studies have shown that PDPN is required for separation of blood and lymphatic vessels during development, because PDPN on lymphatics is engaged by CLEC-2 on platelets and leads to platelet aggregation and vessel separation (Bertozzi et al., 2010). Thus, on most backgrounds, *Pdpn*^−/−^ mice die shortly after birth due to severe defects in lymphatic-vessel formation leading to fatal edema (Bertozzi et al., 2010). PDPN has been reported to be upregulated by dermal fibroblasts exposed to inflammatory mediators. PDPN expression is also increased on tumor cells, believed to promote tumor cell invasion and metastatic spread, and correlated with poor prognosis (Wicki and Christofori, 2007).

CLEC-2 (encoded by *Clec1b*) is a member of the dectin-1 subfamily of C-type lectin receptors (CLRs) and is the receptor for PDPN (Watson et al., 2010). CLEC-2 expression by leukocytes, including human DCs, was reported over a decade ago (Colonna et al., 2000), but its function in these cells has remained enigmatic. CLEC-2 was first discovered on platelets as the receptor that induces aggregation following exposure to the snake toxin rhodocytin. Similar to *Pdpn*^−/−^ mice, *Clec1b*^−/−^ mice are embryonic lethal due to profound defects in vascular development (Suzuki-Inoue et al., 2010). In vitro studies have shown that CLEC-2 plays a role in endocytosis by neutrophils (Kerrigan et al., 2009) and may mediate engulfment of HIV type 1 (Watson et al., 2010). CLEC-2 binds to PDPN via its extracellular C-type lectin-like domain, which promotes CLEC-2 multimerization at the plasma membrane (Watson et al., 2010). CLEC-2 contains a hemi-immunoreceptor tyrosine-based activation motif that has a single YXXL sequence in its cytoplasmic tail. CLEC-2 activation in platelets initiates a tyrosine-phosphorylation signaling cascade mediated by Src, Syk, Vav, SLP-76, and PLCγ family members (Watson et al., 2010). Syk has recently been reported to mediate phosphorylation of CLEC-2 in myeloid cells as well (Mourão-Sá et al., 2011). Vav phosphorylation is required for PLCγ activation in platelets, and CLEC-2-induced platelet aggregation is attenuated in the absence of Vav1 and Vav3 (Pearce et al., 2004). Our results, described below, reveal a role for CLEC-2 in the migratory behavior of DCs, whereby CLEC-2-mediated rearrangement of the actin cytoskeleton is induced by interactions with the PDPN-rich stromal scaffold.

## Results

### DCs Interact Closely with the PDPN-Rich Lymphatic Vessels and FRC Scaffold

Stromal cells provide three-dimensional (3D) scaffolds and molecular cues that coordinate leukocyte migration and compartmentalization within lymphoid organs (Bajénoff et al., 2007). En route to LNs, DCs migrate through lymphatics and along the FRC network to reach the T cell zone. Previous studies have shown that DCs make contact with LECs upon emigrating from peripheral tissues (Baluk et al., 2007; Stoitzner et al., 2002); however, it has not been previously demonstrated whether DCs make direct contact with LECs or FRCs in vivo. Using multiphoton intravital microscopy, we observed that DCs migrating into the LN interacted closely with afferent lymphatic vessels (Figure S1A available online), often for many minutes, before squeezing between collagen fibers and entering the LN (Figure S1B and Movie S1). Electron microscopy further revealed close contact between DCs and FRCs within the subcapsular sinus (Figure S1C).

**Figure 1:**
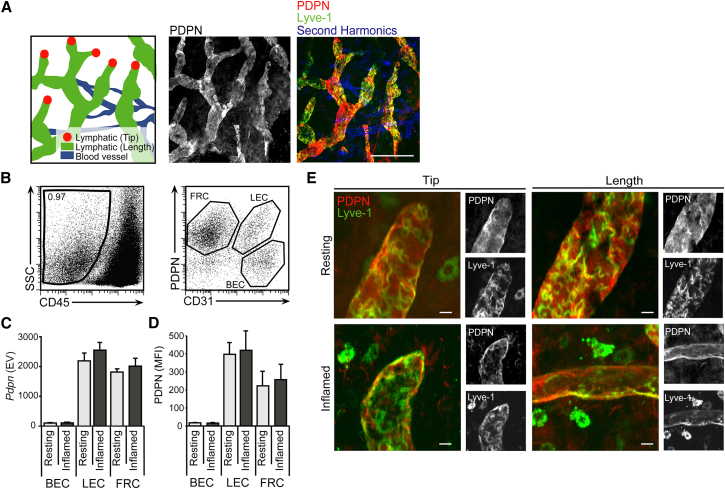
*Pdpn* mRNA and Protein Expression by LECs and FRCs (A) Fluorescence microscopy of tissue whole mounts and schematics of ear skin at resting state. The scale bar represents 100 μm. (B) Representative dot plots showing proportion of stromal cells (CD45^−^) (left panel) and subsets of lymphoid stromal cells (FRCs, CD31^−^PDPN^+^; LECs, CD31^+^PDPN^+^; blood endothelial cells (BECs), CD31^+^PDPN^−^) (right panel) in draining LNs at rest. SSC, side scatter. (C) Graph representing the expression value (EV) of *Pdpn* mRNA from microarray analysis of sorted BECs, LECs, and FRCs from resting or inflamed LNs. (D) Graph showing mean fluorescence intensity (MFI) of surface PDPN on BECs, LECs, and FRCs isolated from draining LNs 24 hr after intradermal injection of PBS (resting) or LPS (inflamed) into ear skin. Data represent mean values and SD from four independent experiments. (E) Fluorescence microscopy of ear skin whole mounts at 0 (resting) and 24 hr (inflamed) after intradermal LPS injection. Fluorescence images show overlays of PDPN (red) and Lyve-1 (green) antibody staining in the tips and along the lengths of afferent lymphatic vessels. Representative black and white images from three independent experiments show individual PDPN and Lyve-1 stains. The scale bar represents 20 μm.

LECs and FRCs, which line the structures that DCs utilize during migration to the T cell zone of the LN, express PDPN at their surfaces (Farr et al., 1992). However, the precise distribution, localization, and abundance of this glycoprotein under steady-state and inflammatory conditions has not been previously described. With the use of whole-mount staining of ear skin, PDPN was found to be expressed both at the tips and along the length of lymphatic vessels, with a mostly uniform distribution (Figure 1A). Using microarray and cytometric analysis of LN stromal cell subsets, we found that PDPN was expressed at the messenger RNA (mRNA) and protein levels by LECs and FRCs, but not by blood endothelial cells (Figures 1B–1D). Furthermore, we found that the amounts of *Pdpn* mRNA and protein and the localization of PDPN on lymphatics were unchanged during inflammation (Figures 1C–1E). These results suggest that PDPN is constitutively expressed by stromal structures in skin, lymphatics, and LNs under resting and inflammatory conditions.

### CLEC-2 Expression by DCs

**Figure 2:**
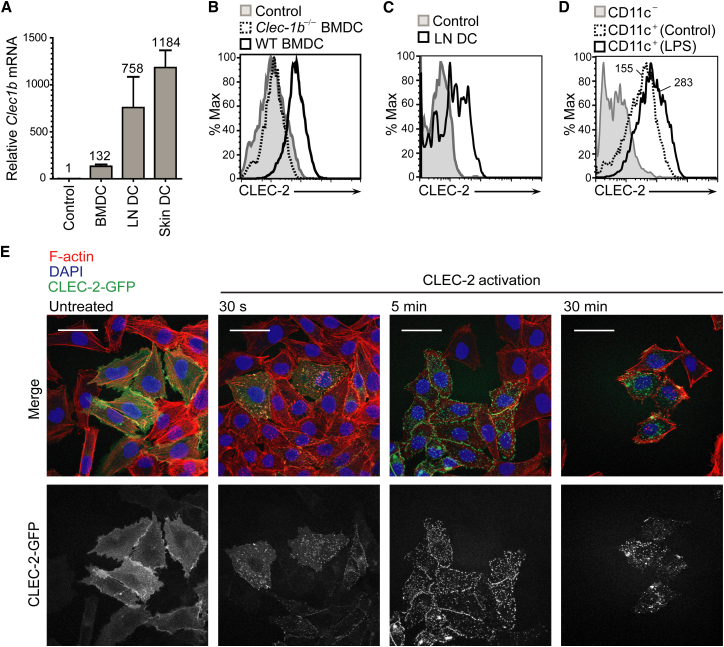
*Clec1b* mRNA and Protein Expression by DCs (A) Quantitative PCR analysis of *Clec1b* mRNA levels in FRCs (negative control), BMDCs, LN DCs, and skin DCs. LN and skin DCs were sorted from primary tissues via flow cytometry. Values above bars depict the mRNA level relative to the negative control. Error bars represent mean and SD for three independent experiments. (B) Flow-cytometry analysis of surface CLEC-2 protein using rPDPN-Fc on WT (solid line) and *Clec1b*^−/−^ (dashed line) BMDCs. The gray line (filled histogram) represents the secondary control. (C) FACS analysis of surface CLEC-2 protein using rPDPN-Fc on freshly isolated WT LN DCs (solid line). Gray line (filled histogram), secondary control. (D) Flow cytometry analysis of surface CLEC-2 protein using rPDPN-Fc on BMDCs treated for 12 hr with LPS (solid line) or left untreated (dashed line). The gray line (filled histogram) represents the secondary control. (E) Representative fluorescence microscopic images from three independent experiments of A375 cells transfected with CLEC-2-GFP that were either stimulated with rhodocytin or left untreated. Top panels, overlay of F-actin (red), DAPI (blue), and CLEC-2-GFP (green). Bottom panels, CLEC-2-GFP fluorescence alone. Scale bars represent 50 μm.

Next, we sought to determine whether the DCs express the PDPN receptor, CLEC-2. CLEC-2 is reportedly expressed by human and mouse platelets and various myeloid cell types, including blood neutrophils, spleen DCs, bone-marrow-derived DCs (BMDCs), and peritoneal macrophages (Bertozzi et al., 2010; Colonna et al., 2000; Kerrigan et al., 2009; Mourão-Sá et al., 2011). Consistent with those reports, we found that *Clec1b* mRNA was expressed by BMDCs (Figure 2A). We also found that *Clec1b* mRNA was expressed by DCs freshly isolated from skin and LNs (Figure 2A). LN and skin DCs expressed ∼750-fold and ∼1,200-fold higher amounts of *Clec1b* mRNA, respectively, than FRCs, which served as a negative control. BMDCs expressed ∼100-fold higher amounts of *Clec1b* mRNA than the negative control. Using flow cytometry with recombinant PDPN-Fc (rPDPN-Fc), which binds specifically to CLEC-2, we confirmed surface expression of CLEC-2 on BMDCs (Figure 2B) and LN DCs (Figure 2C). Thus, DCs from skin, LN, and bone-marrow cultures express CLEC-2 at the mRNA and protein levels.

Next, we examined whether CLEC-2 surface expression by DCs changed upon exposure to an inflammatory mediator. We found that exposure to lipopolysaccharide (LPS) caused CLEC-2 surface expression to increase ∼2-fold (Figure 2D). However, in studies where we monitored the subcellular localization of CLEC-2 using A375 cells transfected with a CLEC-2-green fluorescent protein (GFP) fusion protein, we found that this molecule rapidly clustered at the plasma membrane and then partially internalized in a dynamin-dependent manner into vesicular structures (Figure 2E and data not shown). Thus, CLEC-2 may be upregulated on the DC surface following exposure to an inflammatory stimulus, but in a stromal cell niche where PDPN is constitutively present, some portion of CLEC-2 may be quickly internalized following ligand engagement.

### CLEC-2 Expression Is Required for Efficient DC Migration to LNs

**Figure 3:**
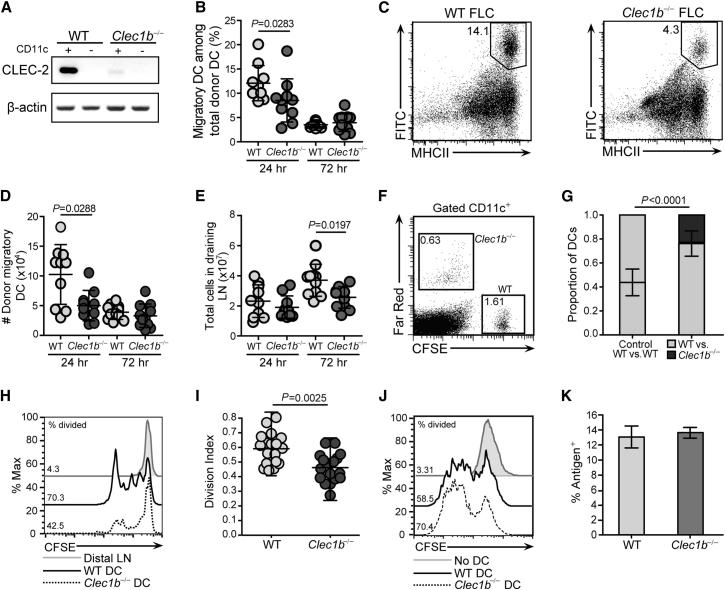
DCs Utilize CLEC-2 for Efficient Migration from Skin to Draining LNs (A) RT-PCR analysis of *Clec1b* mRNA in CD11c^+^ and CD11c^−^ cells that were magnetic-activated cell sorting (MACS)-purified from WT or *Clec1b*^−/−^ FLCs. (B) Percentages of migratory (MHCII^hi^FITC^+^) DCs among total donor (CD45.2^+^) DCs in draining LNs of WT and *Clec1b*^−/−^ FLC mice at 24 and 72 hr post-FITC painting. Error bars represent mean and SD. (C) Representative dot plots (gated on CD45.2^+^CD11c^+^ cells) showing MHCII^hi^FITC^+^ DCs in WT and *Clec1b*^−/−^ FLCs 24 hr after FITC painting. (D) Total numbers of migratory donor (CD45.2^+^CD11c^+^MHCII^hi^FITC^+^) DCs in draining LNs of WT and *Clec1b*^−/−^ FLCs at 24 and 72 hr post-FITC painting. Error bars represent mean and SD. (E) Total cellularity in draining LNs collected from WT and *Clec1b*^−/−^ FLCs 24 and 72 hr after FITC painting. Error bars represent mean and SD. (B, D, and E) Data represent ten mice per experimental condition from three independent experiments. (F) FACS analysis of popliteal (draining) and axillary (distal) LN 24 hr after injection of WT (CFSE^+^) and *Clec1b*^−/−^ (Far red^+^) DCs mixed in equal numbers (2 × 10^5^ of each) prior to injection into the footpad. (G) Quantification of DCs arriving in popliteal LNs 24 hr after footpad injection. Data represent 15 mice per experimental condition from three independent experiments. Error bars represent mean and SD. (H) Histograms showing OT-1 T cell proliferation (CFSE dilution) in popliteal LN following footpad injection of OVA-peptide-loaded WT and *Clec1b*^−/−^ DCs. Numbers show the percentage of divided cells among donor OT-1 T cells. (I) Summary of data, as in (H). Data are shown as a division index. Error bars represent mean and SD. (J) Histograms showing proliferation of OT-1 T cells upon coculture of naive, CFSE-labeled OT-1 T cells with OVA-peptide-loaded WT and *Clec1b*^−/−^ DCs for 48 hr. Numbers indicate the percentage of divided cells among donor OT-1 T cells. Data are representative of three independent experiments. (K) Percentage of WT or *Clec1b*^−/−^ DCs that captured particulate antigen (fluorescently labeled latex beads). Error bars represent mean and SD.

Given that PDPN is expressed along the entire migratory route from skin to LNs and that DCs in these sites express the PDPN receptor, CLEC-2, we tested whether the CLEC-2 pathway contributed to their migration using several approaches. As a first test, we employed fluorescein isothiocyanate (FITC) skin painting to examine migration of skin-resident DCs to LNs in mice lacking CLEC-2. Because *Clec1b*^−/−^ mice exhibit neonatal lethality, fetal liver chimeras (FLCs) were generated from wild-type (WT) and *Clec1b*^−/−^ embryos for evaluation of CLEC-2 function. Six weeks after reconstitution, CLEC-2 expression was analyzed in CD11c^+^ and CD11c^−^ LN cells from WT or *Clec1b*^−/−^ FLCs. As expected, *Clec1b* mRNA signal was detected in CD11c^+^ cells from WT FLCs, but not *Clec1b*^−/−^ FLCs (Figure 3A). Notably, the *Clec1b* mRNA in WT FLCs tracked with CD11c^+^ cells (Figure 3A), suggesting that CLEC-2 is not highly expressed by CD11c^−^ cells in LNs. Ears of WT and *Clec1b*^−/−^ FLCs were painted with FITC, and draining LNs were analyzed via flow cytometry 24–72 hr later. WT migratory DCs (FITC^+^CD11c^+^MHCII^hi^) entered the draining LNs at 24 hr; however, the percentage and total numbers of *Clec1b*^−/−^ DCs were significantly reduced compared with WT DCs (30% and 50%, respectively; Figures 3B–3D). By 72 hr, the numbers of FITC^+^CD11c^+^MHCII^hi^ DCs present in draining LNs had decreased compared with the 24 hr time point, and no differences were observed between the conditions (Figures 3B and 3D). That no significant difference was measured at this time point could reflect a more important function for CLEC-2 in dermal DCs, which migrate to LNs more rapidly than Langerhans cells. However, the dependence of Langerhans cells, which arrive in peak numbers at 72 hr, on CLEC-2 could not be directly evaluated in our FLCs due to their radioresistant nature (Randolph et al., 2005). Consistent with the decrease in *Clec1b*^−/−^ DC migration at 24 hr, LN cellularity was reduced 30% at 72 hr in *Clec1b*^−/−^ compared with WT FLCs (Figure 3E). This difference probably reflects an impaired adaptive immune response resulting from a combination of reduced numbers of migratory DCs arriving at earlier time points and the inability of the *Clec1b*^−/−^ DCs to efficiently enter the T zone and crawl along the FRC scaffold.

As a second test, competitive in situ migration assays with WT and *Clec1b*^−/−^ BMDCs were performed. WT and *Clec1b*^−/−^ DCs were labeled with carboxyfluorescein succinimidyl ester (CFSE) and far-red dyes, respectively, mixed in equal numbers (2 × 10^5^ of each), and coinjected into the same footpad. Donor DCs arriving in the draining (popliteal) and distal LNs were enumerated 24 hr later via flow cytometry. The numbers of *Clec1b*^−/−^ DCs were significantly reduced compared with their WT counterparts, and WT DCs migrated 3-fold more efficiently from skin to LN (Figures 3F and 3G). Similar results were obtained in experiments in which far-red-labeled WT and CFSE-labeled *Clec1b*^−/−^ DCs were used, thereby ruling out any specific effect of the fluorescent dyes on migration (data not shown). As a third approach to confirm the role of CLEC-2 in migration, DCs were treated with rPDPN-Fc protein prior to injection to occupy surface CLEC-2 (see Figure 2B) and thereby outcompete its capacity to bind endogenous PDPN on stroma. Enumeration of migratory DCs in the draining LN revealed that engagement of CLEC-2 with rPDPN-Fc prior to injection significantly reduced their capacity to reach LNs (Figure S2A). Finally, we tested the role of PDPN in DC migration from skin to LN following local injection of short interfering RNA (siRNA). When PDPN expression was reduced on stromal cells, DC migration to the draining LN was reduced by 50% (Figures S2D–S2F).

Our results thus far suggested that efficient migration of DCs requires CLEC-2 binding to PDPN on the surface of stromal cells lining their migratory path. However, another possible explanation was that *Clec1b*^−/−^ DCs displayed intrinsic defects in responsiveness to motility cues, such as CCR7 ligands, or to maturation stimuli. To address this possibility, we first compared the ability of WT and *Clec1b*^−/−^ DCs to migrate toward a CCL21 gradient in a transwell plate. Importantly, both sets of DCs migrated efficiently into the lower chamber in response to the chemokine, and no significant differences were observed (Figure S2G). These data demonstrate that responsiveness to CCR7 ligands is not impaired in *Clec1b*^−/−^ DCs, leading us to conclude that the migration defect observed in *Clec1b*^−/−^ DCs was due to a CCR7-independent signaling pathway directly downstream of CLEC-2. Second, we evaluated the ability of WT and *Clec1b*^−/−^ DCs to mature in response to a proinflammatory stimulus. *Clec1b*^−/−^ BMDCs exhibited the same phenotype as their WT counterparts (Figure S2H) and responded normally to LPS by upregulating surface MHC class II and B7-2 (CD86) (Figure S2I). Thus, the impaired migratory potential of *Clec1b*^−/−^ DCs was not due to intrinsic defects in chemotaxis, nor was it due to maturation.

To determine the functional significance of migratory defects observed in *Clec1b*^−/−^ DCs, we assessed the ability of migratory DCs to stimulate naive T cells in vivo. First, mice received ovalbumin (OVA)-peptide-loaded DCs from WT or *Clec1b*^−/−^ donor mice via footpad injection, followed by intravenously administered naive, CFSE-labeled OT-1 T cells 1 day later. OT-1 T cell proliferation was significantly impaired in mice that received *Clec1b*^−/−^ DCs compared with their WT counterparts (Figures 3H and 3I). Similar results were also observed when WT DCs were preincubated with rPDPN-Fc prior to injection (Figures S2B and S2C).

Second, we evaluated whether the impaired T cell responses with *Clec1b*^−/−^ DCs were due to a difference in their capacity for capturing or presenting antigen. An in vitro T cell activation assay was used to compare the ability of WT and *Clec1b*^−/−^ DCs to stimulate antigen-specific T cells. DCs were pulsed with OVA peptide (SIINFEKL) and then cocultured with naive, CFSE-labeled OT-I T cells. As shown in Figure 3J, OT-I T cells divided to the same extent whether WT or *Clec1b*^−/−^ DCs served as antigen-presenting cells. We then examined whether endocytic activity might explain the different abilities of WT and *Clec1b*^−/−^ DCs to stimulate T cell responses in vivo and found that WT and *Clec1b*^−/−^ DCs exhibited equal antigen-uptake activity (Figure 3K). Thus, *Clec1b*^−/−^ DCs exhibit alterations in migration but appear to capture and present soluble antigens normally. Collectively, these data suggest that CLEC-2 contributes to adaptive immunity by fostering migration of antigen-bearing DCs from tissues to LNs.

### Migratory DCs Use CLEC-2 at Multiple Junctures En Route to LNs

**Figure 4:**
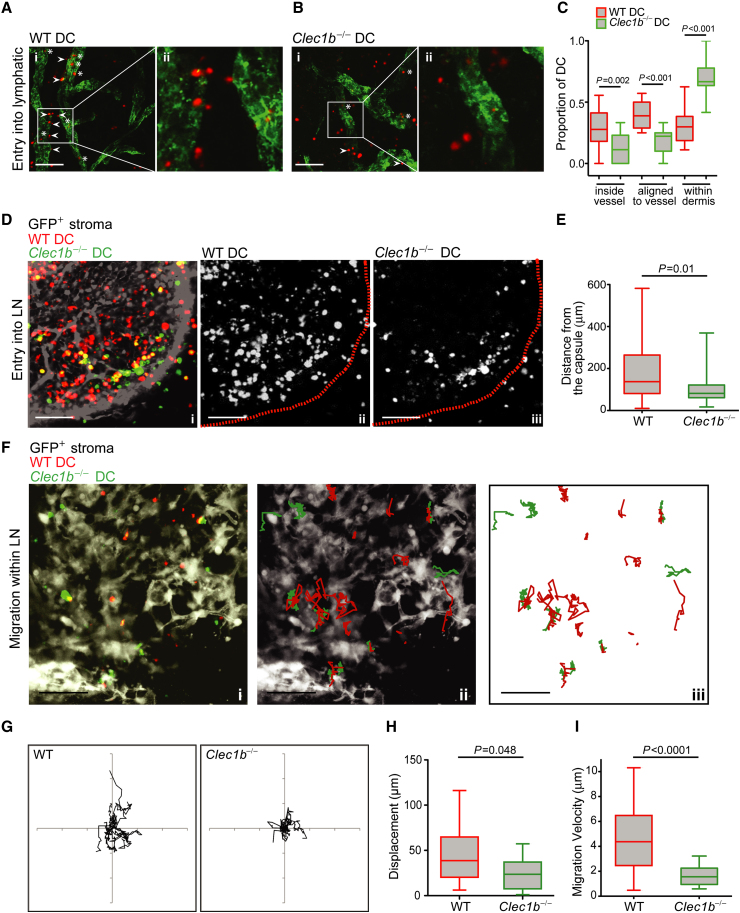
*Clec1b*^−/−^ DCs Exhibit Impaired Migration In Vivo (A) Z projection of ear dermis incubated with WT BMDCs (Ai). Lymphatic vessels are shown in green and infiltrating DCs are shown in red. The scale bar indicates 100 μm. Zoomed image showing DCs interacting with lymphatic vessel (Aii). (B) Z projection of ear dermis incubated with *Clec1b*^−/−^ BMDCS (Bi). The scale bar indicates 100 μm. Zoomed image showing DCs interacting with lymphatic vessel (Bii). (C) Quantification of localization of WT and *Clec1b*^−/−^ DCs within ear sheets. Data are collated from three independent experiments. Error bars represent mean and SD. (D) Z projection of LN from a Ub-GFP BM chimeric mouse (WT BM > Ub-GFP host) injected with WT and *Clec1b*^−/−^ DCs 24 hr prior to dissection and fixation (Di). The GFP^+^ stroma is shown in white, WT DCs are shown in red, and *Clec1b*^−/−^ DCs are shown in green. The scale bar indicates 100 μm. Location of WT DCs relative to LN capsule (dotted line) (Dii). Location of *Clec1b*^−/−^ DCs relative to LN capsule (dotted line) (Diii). (E) Quantification of distance from the LN capsule. Error bars represent mean and SD. (F) Z projection of vibratome-cut GFP-ubiquitin LN slice showing the position of WT (red) and *Clec1b*^−/−^ (green) infiltrated DCs (Fi). GFP^+^ lymph node stromal cells are shown in white. The scale bar indicates 100 μm. Overlay showing tracks of WT (red) and *Clec1b*^−/−^ (green) DCs on GFP^+^ LN (white) over 2 hr period of time-lapse imaging (Fii). Tracks of WT (red) and *Clec1b*^−/−^ (green) DCs within LN slice (Fiii). (G) Representative plots showing directionality of migrating WT and *Clec1b*^−/−^ DCs within LN slices of Ub-GFP BM chimeric mouse with GFP^+^ stroma. Tracks were adjusted to begin at origin (0.0) and overlaid. The axes span 150 μm. (H and I) Displacement (H) and velocity (I) of WT and *Clec1b*^−/−^ DCs within LN slices of Ub-GFP BM chimeric mouse. Data are collated from eight LN slices from four different donors, from three independent experiments. Error bars represent mean and SD.

Having shown that migratory DCs probably encounter PDPN at various points along their migration to LNs, we next determined precisely where the CLEC-2-PDPN interaction played a role. Intravital microscopy of ear skin was employed for evaluation of whether CLEC-2 was important for DC entry into afferent lymphatics. WT DCs rapidly associated with and were able to enter lymphatic vessels (Figures 4A and 4C). In contrast, the majority of *Clec1b*^−/−^ DCs remained in the dermis and failed to interact with lymphatic structures (Figures 4B and 4C). Overall, there was a 30% decrease in the amount of *Clec1b*^−/−^ DCs that entered lymphatics. Furthermore, WT DCs were able to migrate from the subcapsular sinus deep into the LN parenchyma, whereas *Clec1b*^−/−^ DCs were impaired in accessing the paracortex and remained relatively close to the capsule, resulting in a 40% reduction in the distance migrated (Figures 4D and 4E). Finally, we evaluated the motile behavior of WT and *Clec1b*^−/−^ DCs within LNs. Both WT and *Clec1b*^−/−^ DCs penetrated agarose-embedded LNs and showed motile behavior therein (Figure 4F, Movie S2); however, fewer *Clec1b*^−/−^ DCs infiltrated the tissue (data not shown) compared with their WT counterparts, despite equal starting numbers of WT and *Clec1b*^−/−^ DCs. The directionality, displacement, and velocity of DCs that penetrated the tissue were determined over a 120 min period. WT DCs exhibited greater displacement (Figures 4G and 4H) and higher velocity (Figure 4I) compared with *Clec1b*^−/−^ cells, whereas directionality was equivalent among the two DC populations (Figure 4G). Thus, CLEC-2 expression by DCs supports their motility at multiple points along the journey to LNs: from their entry of dermal lymphatics to migration into the LN paracortex.

### CLEC-2 Activation Leads to Protrusion Formation and Stabilization in DCs

**Figure 5:**
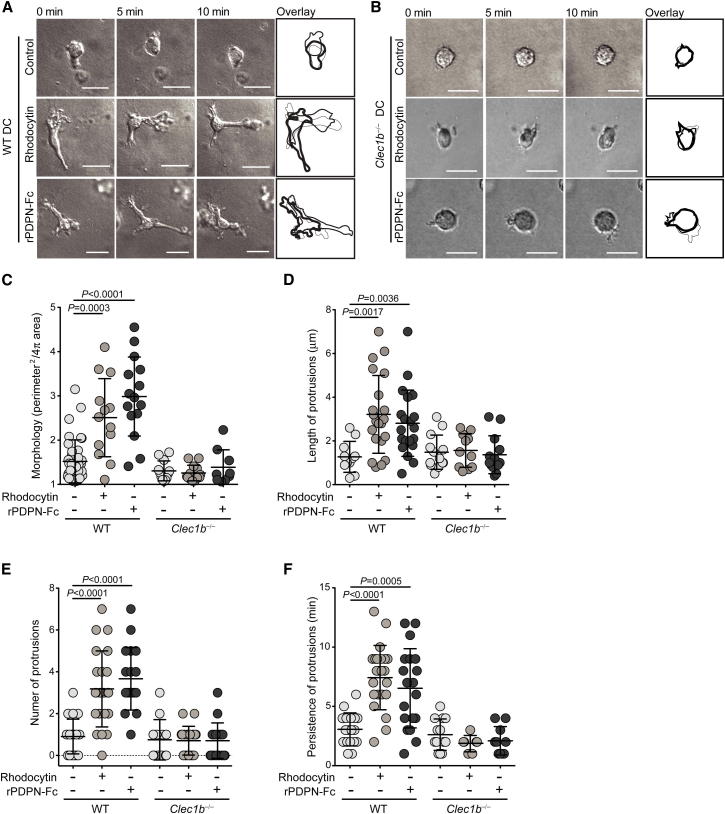
CLEC-2 Activation Induces Protrusion Formation in DCs (A and B) Time-lapse imaging of control-, PDPN-Fc-, and rhodocytin-treated WT (A) and *Clec1b*^−/−^ (B) BMDCs at 0, 5, and 10 min after stimulation. Scale bars represent 20 μm. Far-right panels, overlays of still traces from each time point. (C–F) Morphology index (C), protrusion length (μm) (D), number of protrusions per cell (E), and protrusion persistence (min) (F) for control, rPDPN-Fc, and rhodocytin-treated DCs from WT and *Clec1b*^−/−^ FLCs, as in (A) and (B). Error bars represent mean and SD.

Next, we sought to investigate the mechanism by which CLEC-2 promotes DC migration. Initially, we asked whether CLEC-2 activation might trigger DC maturation, which is known to promote migratory potential, but no activation was observed upon rhodocytin treatment (Figure S3). Time-lapse imaging was then employed to examine the morphodynamic behavior of DCs following CLEC-2 activation. DCs were cultured in 3D collagen matrices (Hooper et al., 2006) following treatment with either rPDPN-Fc or rhodocytin. Notably, CLEC-2 activation triggered vigorous protrusive activity in WT DCs, with multiple, highly branched protrusions extending simultaneously from the cell body (Figure 5A, Movie S3). In contrast, *Clec1b*^−/−^ DCs maintained a rounded morphology following stimulation with either ligand (Figures 5B and 5C), indicating that protrusion activity triggered by rPDPN-Fc or rhodocytin was CLEC-2-dependent. Compared with untreated DCs, which maintained a more rounded morphology and displayed small and short-lived pseudopods (Figures 5A–5C, Movie S3), CLEC-2 ligand-induced protrusions extended greater distances (Figure 5D) from the cell body, were more abundant (Figure 5E), and persisted for longer periods of time (Figure 5F). Thus, CLEC-2 activation triggers dramatic changes in the morphodynamic behavior of DCs, driving the generation and stabilization of cell protrusions.

### The PDPN-CLEC-2 Interaction Is Necessary and Sufficient to Induce DC Protrusions

**Figure 6:**
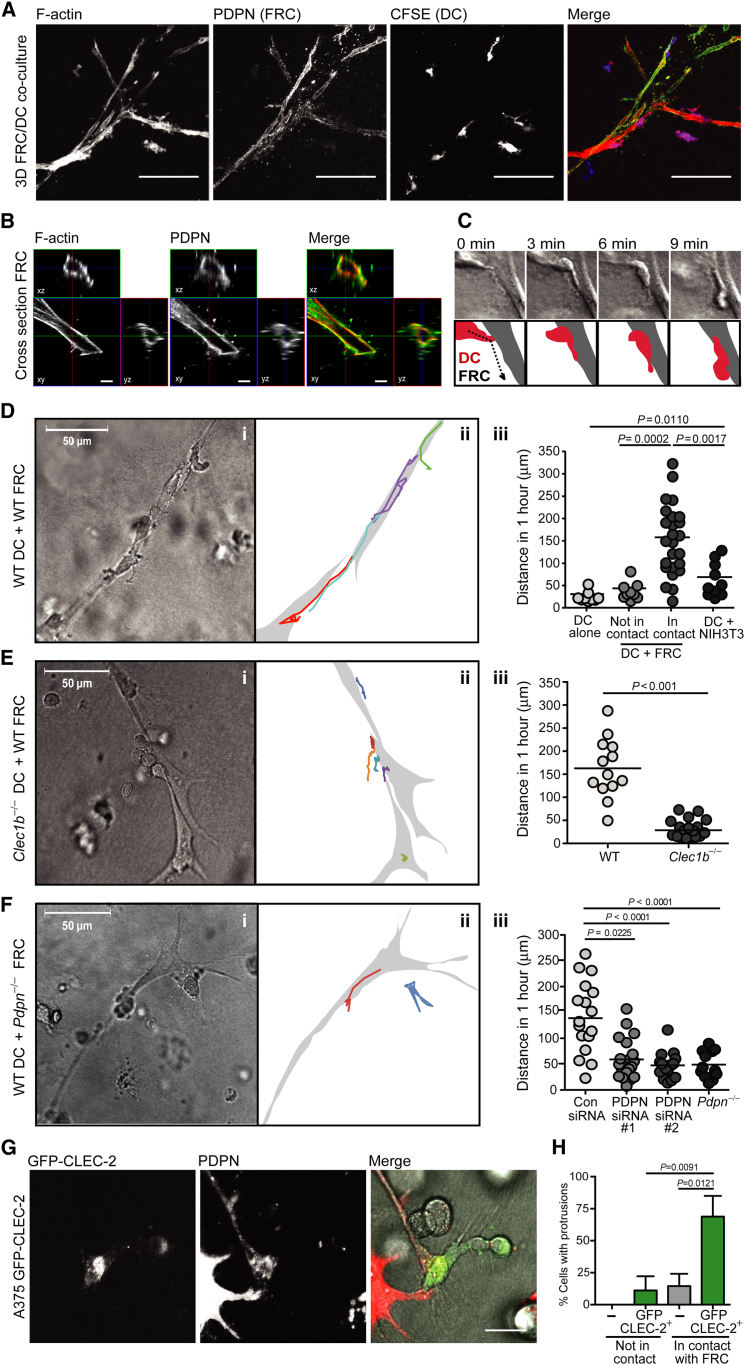
CLEC-2-PDPN Interaction Is Required for DC Migration Along, but Not Attachment to, the FRC Network (A) Z projection of 3D FRC network with DCs. The scale bar represents 100 μm. (B) Representative cross-section of FRC in 3D culture showing PDPN localization at the plasma membrane. The scale bar represents 5 μm. (C) Time-lapse imaging (top) and schematic (bottom) of DC-FRC interaction at 1, 3, 6, and 9 min after contact. (D) Transmitted-light image of WT DCs interacting with WT FRCs in 3D network (Di). The scale bar represents 50 μm. Diagram showing tracks of migrating DCs over 1 hr time course (Dii). Quantification of DC migration along primary FRCs or NIH 3T3 fibroblasts in 3D network (Diii). Each point represents the path of one DC collated from > three independent experiments. (E) Transmitted-light image of *Clec1b*^−/−^ DCs interacting with WT FRCs in 3D network (Ei). The scale bar represents 50 μm. Diagram showing tracks of migrating DCs over 1 hr time course (Eii). Quantification of WT and *Clec1b*^−/−^ DC migration along WT FRCs in 3D network (Eiii). Each point represents the path of one DC. (F) Transmitted-light image of WT DCs interacting with *Pdpn*^−/−^ FRCs in 3D network (Fi). The scale bar represents 50 μm. Diagram showing tracks of migrating DCs over 1 hr time course (Fii). Quantification of WT DCs along FRCs with control siRNA, PDPN-targeted siRNAs, or *Pdpn*^−/−^ FRCs in the 3D network (Fiii). Each point represents the path of one DC. (G) Immunofluorescence showing A375 GFP-CLEC-2 transfectants in contact with FRCs in the 3D network. The scale bar represents 20 μm. Error bars represent mean and SD. (H) Quantification of cells with protrusions, as in (G).

To further dissect the roles of CLEC-2 and PDPN in DC migration along stromal cell scaffolds, we engineered a 3D model of the LN stromal network by seeding primary LN FRCs into a deformable matrix. In 3D, FRCs maintained high expression of PDPN and remodeled the matrix to form an extensively branched, large (50–300 μm in length) reticular structure, reminiscent of the collagen network of the LN paracortex (Figure 6A, compare to Figures 4D–4F). Highly motile leukocytes, such as DCs and B cells, that were coseeded into the networks interacted with the FRC scaffold (Figures 6A and 6B and data not shown). Time-lapse imaging revealed that upon contact with FRCs, DCs extended protrusions and then spread along the stromal cell surface (Figure 6C). Where DCs reached a branch in the FRC scaffold, protrusions were often simultaneously induced along both potential tracks before one was retracted (Movie S4). Notably, PDPN rapidly accumulated at sites of contact between DCs and FRCs (Figure S4A).

Once the DCs adhered to and spread along the FRCs, they began migrating while maintaining close contact with the FRCs. The DCs generally migrated relatively long distances on the FRC network: between 50 and 300 μm in any one direction over 60 min (Figure 6D, Movies S4 and S5). In contrast, DCs not in contact with the FRC network, or cultured alone, were relatively immotile (Figure 6Diii). Interestingly, we found that FRCs isolated from the spleen formed similar networks when seeded into a 3D matrix and also supported DC migration (data not shown). We also evaluated whether any tensile cell scaffold could induce DC migration by creating matrices with NIH 3T3 fibroblasts. When seeded into the NIH 3T3 network, DCs made contact with the fibroblasts but did not migrate efficiently (Figure 6Diii), despite the fact that these cells formed a branched network reminiscent of the FRC scaffolds.

To determine the molecular mechanisms underlying DC migration along FRCs, we first examined the impact of known motility cues. Interestingly, we found that DC migration in the in vitro 3D network occurred independently of CCR7 and its ligands CCL19 and CCL21, as FRCs rapidly downregulated their expression of both CCL19 and CCL21 when cultured ex vivo, and *Ccr7*^−/−^ DCs migrated as efficiently as WT DCs along the scaffold (Figures S4B–S4D). We also tested the requirement of integrins by utilizing FRCs cultured from *Icam1*^−/−^ mice and found, in agreement with published studies, that DCs did not require engagement of this adhesion molecule for migration along stromal networks (Figures S4E–S4G).

Having ruled out a role for CCR7 and ICAM-1 in our 3D model of DC motility, we next directly evaluated whether CLEC-2 activation by PDPN influenced DC migration along FRC scaffolds. To this end, we coseeded 3D matrices with different combinations of DCs and FRCs lacking either CLEC-2 or PDPN and examined DC migration on the networks. When seeded in WT FRC matrices, *Clec1b*^−/−^ DCs were profoundly impaired in migration on the FRC network compared with WT DCs (Figure 6E). In addition to impaired migration, *Clec1b*^−/−^ DCs also failed to spread along the stromal cell surface, exhibiting a distinctly rounded morphology (Figure 6E and Movie S6). These results suggest that DCs still make contact with the FRC network in the absence of CLEC-2 but lack the ability to spread and crawl on this surface.

We also examined the role of PDPN in DC migration along stromal scaffolds. Initially, we compared the motility of WT DCs in networks formed by NIH 3T3 fibroblasts or FRCs. As shown in Figure 6Diii, DCs did not migrate efficiently on the NIH 3T3 fibroblasts compared with FRCs. Interestingly, NIH 3T3 cells express 80% less PDPN than cultured FRCs (Figure S4H), which led us to further examine the sufficiency of PDPN on stroma in this interaction. To this end, we took a gain-of-function approach by overexpressing cyan fluorescent protein (CFP)-PDPN in NIH 3T3 cells to ascertain whether elevated levels of this protein would incite DC migration. Expression of CFP-PDPN in these cells caused morphologic changes that were consistent with previous reports linking PDPN expression with increased invasiveness in cancer cells (Wicki and Christofori, 2007). The CFP-PDPN^+^ NIH 3T3 cells no longer formed extensive networks; however, they did exhibit a stellate morphology when coseeded with DCs in the 3D matrices. Interestingly, we observed enhanced interactions between DCs and CFP-PDPN^+^ NIH 3T3 cells compared with the control NIH 3T3 cells (data not shown). Furthermore, consistent with a requirement for CLEC-2 activation by PDPN in this niche, we found that DC migration was severely diminished in networks formed by *Pdpn*^−/−^ FRCs compared with WT FRCs (Figure 6F, Movie S7, and Figure S4I). Similarly, WT DCs were markedly less efficient at spreading and migrating on FRCs in which PDPN surface levels were reduced following transfection with PDPN-targeting siRNAs compared with control siRNA-transfected FRCs (Figure 6Fiii and Figure S4J). Thus, our findings suggest that PDPN is required for DCs to spread and migrate efficiently along the FRC surface.

To investigate whether CLEC-2 activation was sufficient to induce protrusion formation, we utilized A375 cells, a human melanoma cell line lacking CLEC-2 expression that migrates in an amoeboid manner and exhibits a rounded morphology (Pinner and Sahai, 2008). Control A375 cells that were seeded into the 3D matrix maintained their rounded morphology and did not form protrusions, whether they were in contact with FRCs or not (Figures 6G and 6H). In contrast, A375 cells expressing GFP-CLEC-2 formed long protrusions only when in contact with FRCs (Figures 6G and 6H). These results demonstrate that overexpression of CLEC-2 by a cell type that normally lacks it is sufficient to incite the formation of protrusions in response to contact with PDPN-rich FRCs. Therefore, PDPN activation of CLEC-2 is necessary and sufficient to induce dynamic protrusions and spreading to support migration of DCs, and potentially any CLEC-2-expressing cell type, along PDPN-rich stromal scaffolds.

### CLEC-2 Signaling Coordinately Reduces Actomyosin Contractility and Promotes Actin Polymerization

**Figure 7:**
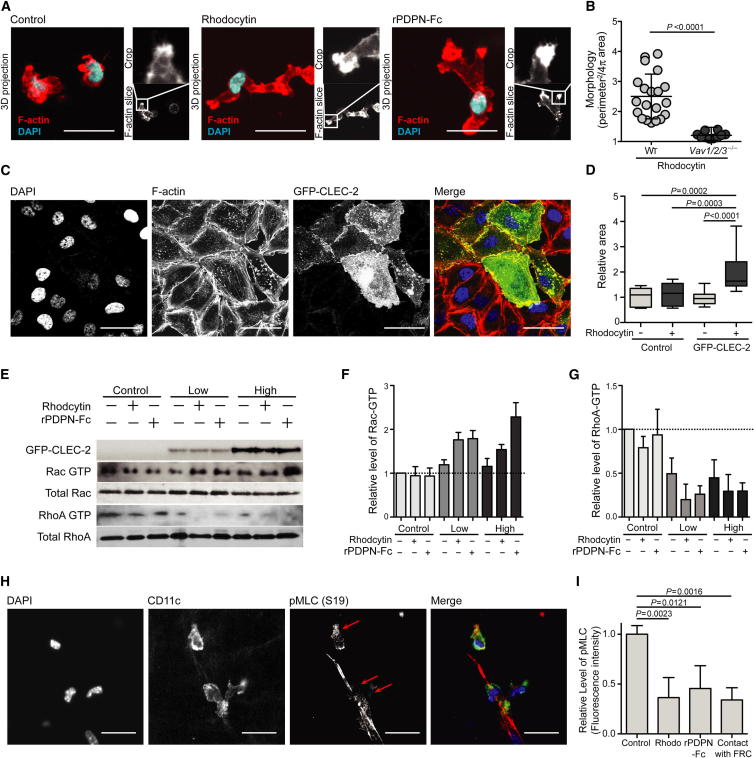
CLEC-2 Signaling Coordinately Reduces Actomyosin Contractility and Promotes Actin Polymerization (A) F-actin and DAPI staining of control, rhodocytin, and rPDPN-Fc-treated DCs. The scale bar represents 20 μm. (B) Morphology index of WT and *Vav1*^−/−^*Vav2*^−/−^*Vav3*^−/−^ DCs. Data are collated from three individual experiments, and each data point represents the morphology of an individual DC. Error bars represent mean and SD. (C) Immunofluorescence of A375 cells transfected with GFP-CLEC-2 and treated with rhodocytin in 2D culture. DAPI, F-actin, and GFP fluorescence are shown. The scale bar represents 20 μm. (D) Quantification of area comprised by cells plated in 2D following rhodocytin stimulation relative to untransfected A375 cells. Error bars represent mean and SD. (E) Protein blots showing levels of GFP-CLEC-2 and total and activated Rac1 and RhoA in A375 clones expressing either low or high levels of GFP-CLEC-2 or A375 control cells following treatment with rPDPN-Fc or rhodocytin. (F) Quantification of Rac1-GTP, as in (G) (n = 3). (G) Quantification of RhoA-GTP, as in (G) (n = 3). Error bars represent mean and SD. (H) Immunofluorescence of FRCs and DCs in 3D network showing DAPI, CD11c, and pMLC (S19) staining. The scale bar represents 20 μm. (I) Quantification of pMLC fluorescence intensity in 3D network relative to untreated DCs or DCs not in contact with FRCs. Error bars represent mean and SD.

To elucidate the mechanism by which CLEC-2 activation promotes cell motility, we investigated how engagement by its ligands affects the actin cytoskeleton. CLEC-2 activation by either rPDPN-Fc or rhodocytin led to a marked accumulation of F-actin in the tips of newly extended protrusions, suggesting that actin polymerization drives their formation (Figure 7A). CLEC-2 signaling has been shown to activate a cascade of tyrosine-kinase signaling involving Vav (Pearce et al., 2004). Given that Vav family members can also function as exchange factors for Rho family GTPases (Graham et al., 2009), master regulators of the actin cytoskeleton (Nobes and Hall, 1995), we investigated whether the morphologic changes in DCs upon CLEC-2 activation were dependent on Vav. Whereas WT DCs exhibited extensive protrusions upon stimulation with rhodocytin, *Vav1*^−/−^*Vav2*^−/−^*Vav3*^−/−^ DCs maintained a rounded morphology upon CLEC-2 activation (Figure 7B). Indeed, the morphology of the *Vav1*^−/−^*Vav2*^−/−^*Vav3*^−/−^ DCs was highly reminiscent of the *Clec1b*^−/−^ DCs shown in Figure 5B (data not shown). These data indicate that the cytoskeletal rearrangements and actin-rich protrusions formed in DCs upon CLEC-2 activation are driven by a similar cascade of tyrosine-kinase signaling, dependent on Vav, as has been described in CLEC-2-induced platelet aggregation (Watson et al., 2010).

The small G protein Rac1 is known to promote the nuclearization and polymerization of actin filaments, and its activation is classically characterized by the spreading of lamellipodia and induction of membrane ruffles (Nobes and Hall, 1995; Olson and Sahai, 2009). Furthermore, rhodocytin activation of CLEC-2 in platelets has been shown to activate Rac1 (Watson et al., 2010). To examine whether the cytoskeletal changes that we observed following CLEC-2 activation by PDPN were indicative of Rac1 activation, GFP-CLEC-2^+^ A375 cells were treated with CLEC-2 ligands and examined in two-dimensional (2D) culture. A375 cells expressing GFP-CLEC-2 spread rapidly upon exposure to rhodocytin and within 5 min had spread to cover nearly twice the surface area of neighboring untransfected cells (Figures 7C and 7D). To directly ascertain whether CLEC-2 signaling triggers actin polymerization via Rac1, we measured the levels of guanosine triphosphate (GTP)-bound Rac1 relative to total Rac1 by protein blot following exposure of GFP-CLEC-2 A375 transfectants to either rPDPN-Fc or rhodocytin. These experiments demonstrated that Rac1 activity was indeed increased as a result of CLEC-2 activation (Figures 7E and 7F). Together, these results suggest that CLEC-2 signaling, initiated by ligand engagement, triggers actin polymerization and protrusion formation via Rac1.

Next, we sought to understand how CLEC-2 activation promotes spreading along the stromal cell surface. RhoA is known to induce myosin light chain (MLC) phosphorylation and increase actomyosin contractility through its interaction with Rho kinases (Parri and Chiarugi, 2010). We wondered whether CLEC-2 signaling may alter RhoA activity to reduce actin contractility and thereby permit a DC or other CLEC-2-expressing cell to spread along the stromal cell surface. To this end, we quantified the amounts of GTP-bound RhoA relative to total RhoA in GFP-CLEC-2^+^ A375 cells that were treated with either rPDPN-Fc or rhodocytin. CLEC-2 activation with either ligand caused a striking reduction in GTP-bound RhoA compared with untreated cells (Figures 7E and 7G), suggesting that the downstream signaling that controls contractility of the actomyosin cytoskeleton would also be reduced as a result of CLEC-2 activation. We directly tested whether this signaling pathway downstream of CLEC-2 might elicit a reduction in contractility of the actin cytoskeleton in DCs. MLCII is a key subunit of the conventional myosin motor, and phosphorylation of MLC at serine 19 (S19) promotes its activity (Wyckoff et al., 2006). The phosphorylation of MLC is highly dependent on the activity of RhoA effector kinases, and the global levels of phosphorylated MLC (pMLC) (S19) in a cell can be used as a measure of the overall MLCII activity and contractility of the actin cytoskeleton (Wyckoff et al., 2006). Thus, we examined pMLC in DCs crawling along stromal cell scaffolds, where CLEC-2 will be activated by engagement of PDPN. Immunofluorescence analysis of 3D DC-FRC cocultures revealed that DCs in contact with the FRC network exhibited markedly reduced levels of pMLC compared with DCs that were not in contact with FRCs (Figures 7H and 7I). Similar measurements were taken in DCs cultured alone in a 3D matrix and treated with CLEC-2 ligands. A significant reduction in the levels of pMLC (S19) in DCs was also observed following CLEC-2 activation by either rhodocytin or rPDPN-Fc (Figure 7I). Collectively, these findings suggest that CLEC-2 engagement by PDPN leads to a coordinate set of events: (1) the upregulation of Rac1 activity and stimulation of actin polymerization to drive protrusion formation, and (2) the downregulation of RhoA activity and a decrease in pMLC to reduce cell contractility. Together, these coordinated events would allow cells to spread along a stromal cell scaffold, extend actin-rich protrusions, and migrate.

## Discussion

Here we describe a role for CLEC-2 signaling in DC motility. The CLEC-2 ligand PDPN is highly expressed by LECs and FRCs lining structures that DCs encounter during migration from tissues to LNs. CLEC-2 is expressed by skin and LN DCs, but surface levels of this protein appear to be tightly regulated. Engagement of CLEC-2 by PDPN coordinately reduces actomyosin contractility and promotes actin polymerization in DCs, thereby allowing them to spread along stromal cell scaffolds, extend protrusions, and migrate. The CLEC-2-PDPN interaction promotes DC migration to LNs at all stages of this journey: from leaving peripheral tissues and entering lymphatic vessels, to crossing the subcapsular sinus, and finally to migrating through LN parenchyma to the T cell zone. T cell activation in LNs was markedly reduced despite the fact that some *Clec1b*^−/−^ DCs completed the passage from skin to LN, as measured by flow cytometry. This could suggest a preferentially important role for CLEC-2 in a DC subset that efficiently cross-presents antigens such as the CD103^+^ dermal DC. Alternatively, this could suggest that CLEC-2 deficiency not only reduces DC migration but also prevents DCs from properly interacting with the FRC network and T cells. Consistent with this latter possibility, we found that *Clec1b*^−/−^ DCs were severely impaired in spreading and crawling on the FRC network in 3D.

Analysis of the downstream signaling components revealed that CLEC-2 activation in DCs, as in platelets, required the adaptor protein and exchange factor Vav and led to alterations of actin dynamics via activation of Rac1. Our study shows that CLEC-2 activation by PDPN induces F-actin-rich protrusions conducive to cell migration. Additionally, we find that RhoA activity is coordinately decreased upon CLEC-2 activation. Rac and Rho have been shown to be antagonistic in a variety of contexts (Ohta et al., 2006; Wildenberg et al., 2006), and this exclusivity may act to spatially separate Rho and Rac. One potential antagonistic mechanism is the ability of Rac to induce the translocation of p190RhoGAP to locally downregulate Rho activity (Wildenberg et al., 2006). An opposing mechanism is also reported whereby Rho activity leads to the activation of FilGAP for inactivation of Rac (Ohta et al., 2006).

Based on the signaling events following contact between *Clec1b*^+^ DCs and *Pdpn*^+^ stromal cells, we propose the following model. Initially, CLEC-2 and PDPN interact and cluster on the respective cell membranes. CLEC-2 oligomerization then induces tyrosine-phosphorylation events via Syk, PLCγ, and Vav. Vav promotes the activity of Rac1, which indirectly and coordinately reduces RhoA activity. These events are spatially restricted to domains on the DC surface that are in direct contact with PDPN. Therefore, specifically at the contacts between DCs and FRCs, the activity of RhoA effectors, such as ROCK (Rho-associated protein kinase), are reduced, leading to reduced phosphorylation of MLC (Wyckoff et al., 2006) and reduced contractility of the actomyosin cytoskeleton. We suggest that this mechanism can explain the ability of DCs to spread along the underlying stromal cell scaffold. The increase in Rac1 activity then promotes actin polymerization and the induction of protrusions reaching along the stromal cell. As the DC reaches forward, CLEC-2 signaling would need to be tempered to allow RhoA effector activity to contract the cell rear and allow the cell body to follow the protrusion (Nobes and Hall, 1995). We have noted that CLEC-2 oligomers are rapidly internalized following ligand binding, which may allow temporal and spatial restriction of CLEC-2 signaling.

The utilization of our 3D coculture system provided critical insights into leukocyte interactions with FRCs and molecular mechanisms promoting DC migration in this stromal niche. For example, by using this system in which primary FRCs downregulate chemokine expression, we discovered that PDPN-driven activation of CLEC-2 was necessary and also sufficient to induce bona fide DC migration in the absence of additional motility cues, such as CCL19 and CCL21. DC migration in this system was confined to FRC surfaces but was not directional. DCs migrated back and forth along FRC scaffolds, suggesting that PDPN activation of CLEC-2 would not be sufficient to guide DCs in one particular direction. Rather, simultaneous signals from chemokine gradients and fluid flow in vivo would confer directionality on DC motility. Coordination of these different signaling processes would thereby endow DCs with the capacity to migrate through complex stromal niches from their sentry posts in parenchymal tissues to tissue-draining LNs where T cell responses commence.

CLEC-2 activation may affect the motility of other leukocytes that express this CLR. Neutrophils have been reported to express CLEC-2 (Kerrigan et al., 2009), and as such, their migration to infected tissues or to tertiary lymphoid structures may also be affected upon CLEC-2 engagement of PDPN. B cells, which also express CLEC-2 (Mourão-Sá et al., 2011; data not shown), may utilize this pathway to migrate along PDPN-rich FRCs en route to follicles in secondary lymphoid organs. Indeed, we found that, like DCs, B cells migrate along FRCs in the 3D culture system (Fletcher et al., 2011) and may use CLEC-2 to move within LNs (data not shown).

Beyond LECs and LN FRCs, PDPN is expressed by splenic FRCs (Bekiaris et al., 2007) and stromal cells in bone marrow and thymus (data not shown). PDPN is also upregulated in tissue fibroblasts during inflammation, and PDPN-rich stroma is often observed in tumors, perhaps supporting the function of tumor-infiltrating leukocytes (Wicki and Christofori, 2007). Activation of CLEC-2 by PDPN may therefore be a widely applicable mechanism promoting leukocyte motility along stromal networks of all PDPN-rich lymphoid organs and inflamed tissues, irrespective of which chemokine cues are present. Thus, targeting CLEC-2 to specifically harness leukocyte migration may have significant therapeutic potential for treating inflammatory and autoimmune diseases.

## Experimental Procedures

### Isolation and Culture of Primary Cells

LN FRCs were isolated and expanded as previously described (Fletcher et al., 2011). For BMDCs, bone marrow was taken from the tibias and femurs of 5-week-old C57BL/6, *Ccr7*^−/−^, *Vav1*^−/−^*Vav2*^−/−^*Vav3*^−/−^, and *Clec1b*^−/−^ mice and cultured in RPMI containing 10% fetal bovine serum and 3% granulocyte macrophage colony-stimulating factor (derived from supernatant of J558L culture) for 5–6 days. Unstimulated BMDCs were used in functional experiments unless otherwise specified. For experiments in which matured BMDCs were used, the cultures were treated with 10 ng/ml LPS for 24 hr.

### 3D Culture System

DCs were seeded either alone or at a 5:1 ratio with FRCs into a collagen/matrigel matrix (Pinner and Sahai, 2008) (1.8 mg/ml Matrigel [BD Biosciences] and 3.2 mg/ml collagen type 1 [BD Biosciences]) on glass-bottomed cell-culture plates (MatTek). Gels were incubated at 37°C with 10% CO_2_ in minimum essential medium alpha containing 10% fetal calf serum (FCS) during imaging. For experiments involving CLEC-2 activation in 3D culture, CLEC-2 ligands (rhodocytin, 200 nM and rPDPN-Fc, 0.04 μg/ml) were added to the gel as all components were mixed.

### Quantification of Cell Morphology and Cell Migration In Vitro

Using ImageJ software, we determined the area and perimeter of cells by manually drawing around the cell shape using phase-contrast images. Ratios were calculated as follows: perimeter^2^/4πarea. The elongation factor was calculated with the ImageJ “fit ellipse” function. The ratio of major/minor axis was plotted for measuring cell elongation on FRCs. ImageJ was also used to determine the cell centroid and its translocation over time to calculate distance migrated along. The motion analysis (Figure S4B) shows three time points, overlaid in red, green, and blue. Static cells and the collagen matrix are shown in white; motile cells are visualized by the separation of the red, green, and blue channels.

### Analysis of DC Movement in Tissues

DC entry to lymphatic vessels was carried out as previously described (Lämmermann et al., 2008). In brief, ears of C57BL/6 mice were split, and the cartilage-free half was incubated with directly conjugated Lyve-1-488 antibody (eBioscience) in PBS containing 1% BSA for 1 hr on ice. The stained ears were incubated with either WT or *Clec1b*^−/−^ LPS-matured BMDCs labeled with 5 mM far-red cell-tracker dye (Invitrogen) for 2 hr at 37°C with 5% CO_2_. Noninfiltrating DCs were then gently washed away, and ears were fixed in 1% paraformaldehyde (PFA) for 4 hr. Ear sheets were imaged with an inverted Zeiss 510 confocal microscope.

For DC migration in LNs, LNs were dissected from Ubiquitin-GFP chimeric mice (WT BM > Ub-GFP host) and embedded in 4% low-melting-temperature agarose at 36°C. Blocks were then cooled on ice. Vibratome sections of LNs 300 μm thick were cut and incubated with RPMI containing 10% FCS for 30 min at 37°C. WT and *Clec1b*^−/−^ BMDCs were labeled with cell-tracking dyes, washed with PBS, then incubated with the LN slices for 90 min at 37°C. After this time, LN slices were gently washed with RPMI containing 10% FCS, laid into MatTek dishes, and imaged with an inverted Zeiss 510 confocal microscope for 2 hr at 37°C with 5% CO_2_. Z stacks of 30 μm were projected with ImageJ Z Project. Time-lapse sequences were analyzed with the ImageJ manual-tracking plugin to measure cell velocity and to track individual cells.

### Statistical Analysis

Statistical tests in this paper were performed with Prism software (GraphPad). Data sets were first subjected to an F test to establish whether data to be compared exhibited comparable SD. Following this, the appropriate tests were performed as indicated in each figure legend. In general, comparison of multiple groups was performed with the use of a Kruskal-Wallis ANOVA and, if significant, followed by Dunn’s post test between individual data sets. Comparisons of two data sets were most commonly performed with the use of a Mann-Whitney U test.

## Supplemental Information

Document S1. Figures S1–S4 and Supplemental Experimental Procedures

Movie S1. Multiphoton Time-Lapse Imaging Shows Migratory DCs Entering the Popliteal Lymph Node as Shown in Figure S4B CFSE-labeled DCs (green), second harmonic (collagen fibers) (red). Frames are 30 s apart and shown at 8 frames/s.

Movie S2. Confocal Time-Lapse Imaging of DC Motility in LN Slices from WT BM → Ub-GFP Mice The DCs were labeled with cell-tracker dyes; WT DCs are shown in red and *Clec1b*^−/−^ DCs are shown in green. Frames are 90 s apart and shown at 8 frames/s.

Movie S3. Time-Lapse Imaging Shows the Behavior of DCs Cultured in 3D Deformable Matrix and Stimulated with CLEC-2 Ligands Control DCs are shown first, followed by DCs treated with rhodocytin and PDPN-Fc. Frames are 90 s apart and shown at 8 frames/s. Scale bar represents 20 μm.

Movie S4. Time-Lapse Imaging Shows DCs Approaching Branched FRCs in 3D Matrix Frames are 90 s apart and shown at 8 frames/s.

Movie S5. Time-Lapse Imaging Shows the Migration of WT DCs along WT FRCs Cultured in 3D Matrix Frames are 2 min apart and shown at 8 frames/s.

Movie S6. Time-Lapse Imaging Shows the Interaction of *Clec1b*^−/−^ DCs with WT FRCs Cultured in 3D Matrix Frames are 2 min apart and shown at 8 frames/s.

Movie S7. Time-Lapse Imaging Shows the Interaction of WT DCs with *Pdpn*^−/−^ FRCs Cultured in 3D Matrix Frames are 2 min apart and shown at 8 frames/s.
