# Supplementary material for: Podoplanin-Rich Stromal Networks Induce Dendritic Cell Motility via Activation of the C-type Lectin Receptor CLEC-2
Source: Immunity. 2012 Aug 24;37(2):276–89. doi: 10.1016/j.immuni.2012.05.022 (PMC3556784; doi:10.1016/j.immuni.2012.05.022)
Supplement: Document S1. Figures S1–S4 and Supplemental Experimental Procedures [file mmc1.pdf]

Immunity, Volume *37*

Supplemental Information

Podoplanin-Rich Stromal Networks Induce Dendritic Cell Motility via Activation of the C-type Lectin Receptor CLEC-2

Sophie E. Acton, Jillian L. Astarita, Deepali Malhotra, Veronika Lukacs-Kornek, Bettina Franz, Paul R. Hess, Zoltan Jakus, Michael Kuligowski, Anne L. Fletcher, Kutlu G. Elpek, Angelique Bellemare-Pelletier, Lindsay Sceats, Erika D. Reynoso, Santiago F. Gonzalez, Daniel B. Graham, Jonathan Chang, Anneli Peters, Matthew Woodruff, Young-A Kim, Wojciech Swat, Takashi Morita, Vijay Kuchroo, Michael C. Carroll, Mark L. Kahn, Kai W. Wucherpfennig, and Shannon J. Turley

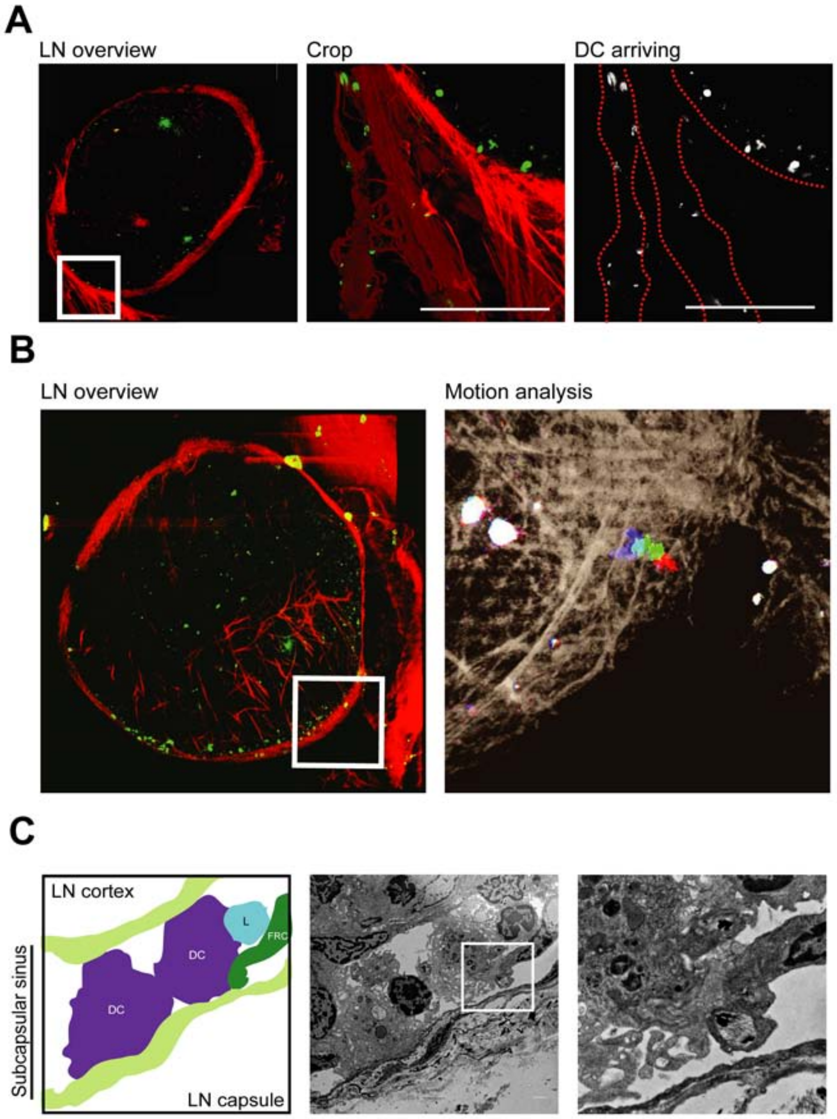

Figure S1. **Migrating DCs Make Direct Contact with Lymphatic Endothelial Cells and FRCs upon LN Entry**

(A) Multiphoton imaging showing migratory DCs interacting with afferent lymphatic vessels. *Far left,* Overview of whole popliteal LN (CFSE-labeled DCs [green], second harmonic (collagen fibres) [red]. White box indicates region shown at higher magnification. *Far right,* schematic showing DC [white] in relation to lymphatic vessels [dotted lines].

(B) Multiphoton imaging showing migratory DCs entering LN. *Left,* Overview of whole popliteal LN (CFSE-labeled DCs [green], second harmonic (collagen fibres) [red]. *Right,* Motion analysis showing path of migratory DC. Sequential time points are overlaid in red, green and blue. Static objects are shown in white, motile objects are shown by the separation of red, green and blue.

(C) *Left,* Schematic showing positions of subcapsular sinus fibroblasts, DCs, a lymphocyte (L), and an FRC. *Right*, transmission electron microscopy of subcapsular sinus; box indicates region shown at higher magnification.

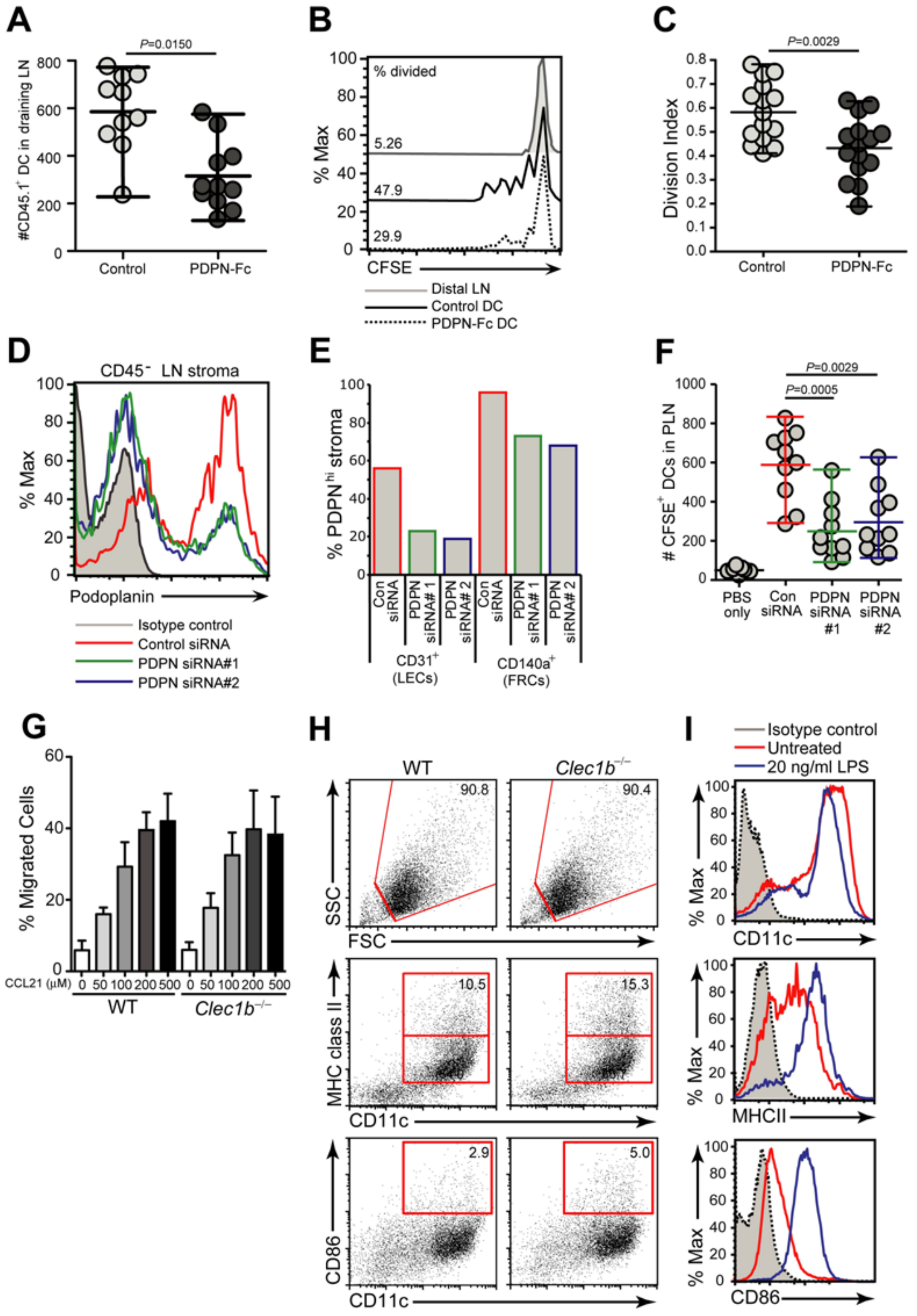

Figure S2. Inhibiting the CLEC-2-PDPN Interaction Impairs DC Migration independently of responses to chemokine gradients

(A) Numbers of control and rPDPN-Fc-coated ($CD45.1^+$) DCs arriving in popliteal LN 24 hrs following footpad injection. Data represent 25 mice per experimental condition collated from 3 independent experiments.

(B) Histograms showing OT-1 T cell proliferation (CFSE dilution) in popliteal LN following footpad injection of OVA-loaded DCs with or without pre-incubation with rPDPN-Fc. Numbers show the percentage of divided cells among donor OT-1 T cells.

(C) Summary of data as in *B* from 15 individual mice per experimental condition and 2 independent experiments. Data are shown as division index.

(D) PDPN expression in stomal cells ($CD45^-$) from popliteal LNs 72 hours following footpad injection of PDPN siRNA.

(E) Quantification of PDPN knockdown in endothelial cells and FRCs in popliteal LNs.

(F) Quantification of reduction in numbers of CFSE-labeled DCs arriving in popliteal LN from footpad following PDPN siRNA injection.

(G) Quantification of DC migration in response to CCL21 concentrations (50-500 μM) using a transwell assay. Data represent mean and standard deviation from 3 independent experiments.

(H) FACS plots showing FSC vs. SSC profiles and surface CD11c, MHC class II, and CD86 (B7-2) on untreated WT and *Clec1b*$^{-/-}$ DCs from day 5 GM-CSF bone marrow cultures.

(I) Histograms showing surface CD11c, MHC class II and CD86 on untreated and LPS-treated DCs from *Clec1b*$^{-/-}$ bone marrow cultures.

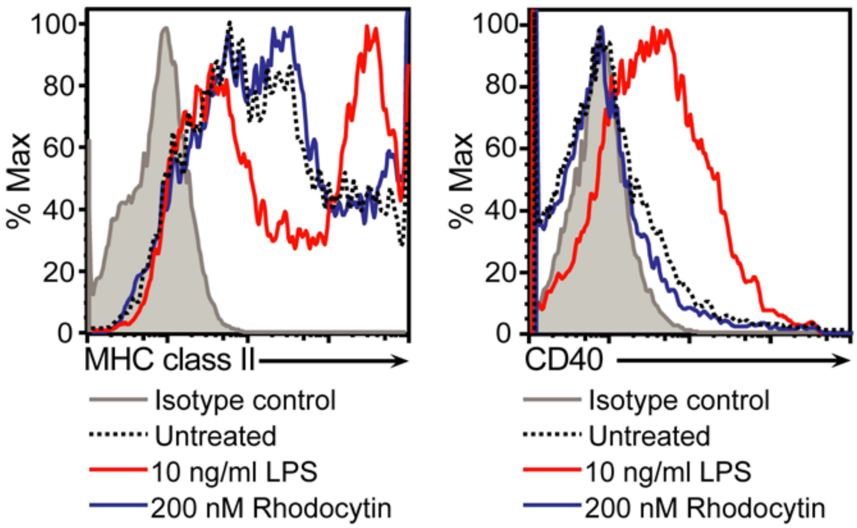

Figure S3. **CLEC-2 Activation Does Not Affect Maturation in DCs**

FACS analysis of surface MHC class II and CD40 levels on DCs 24 hrs following treatment with LPS or rhodocytin.

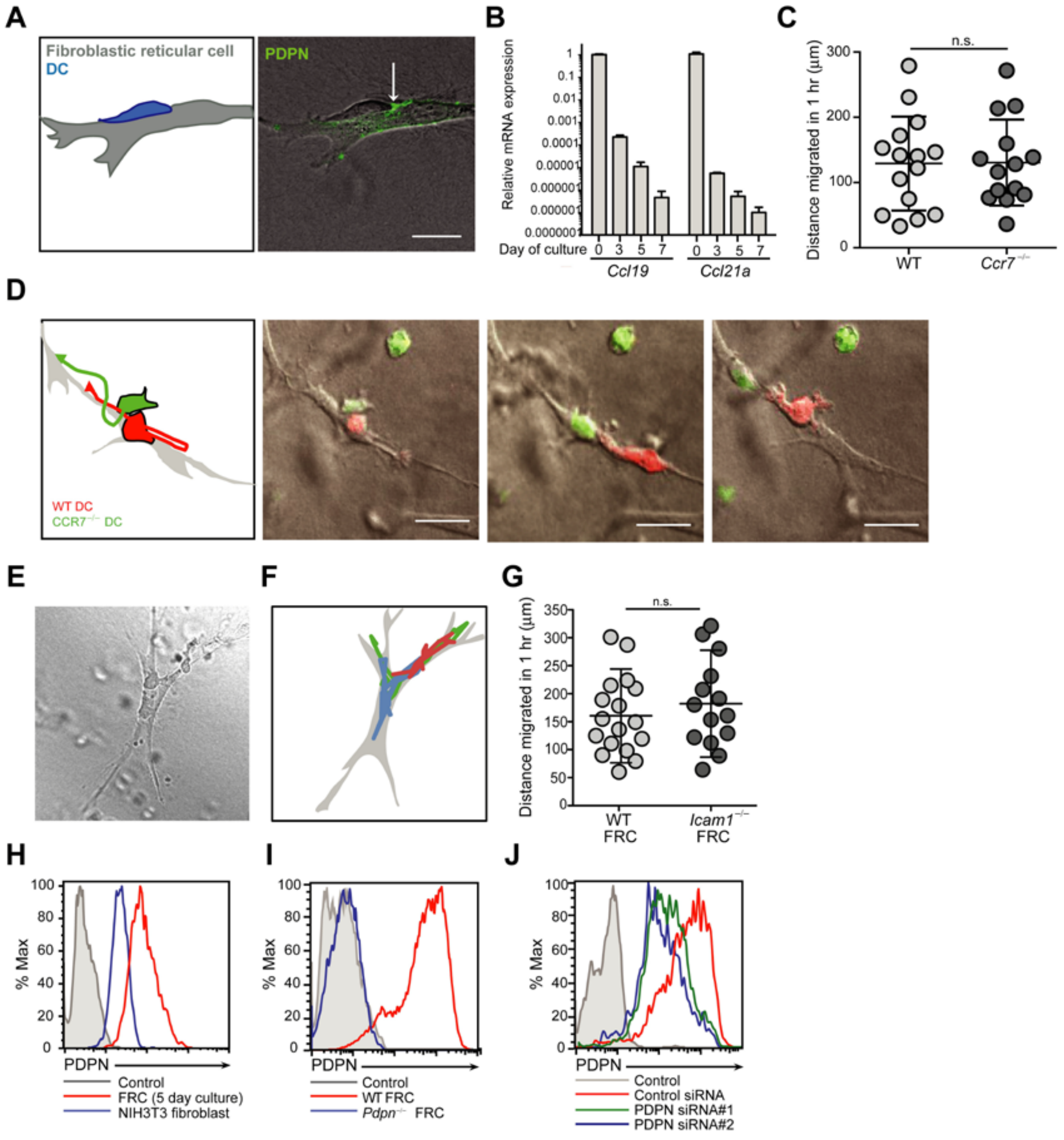

Figure S4. PDPN and CLEC-2 Interact to Direct DC Migration along the FRC Network Independently of CCR7 and ICAM Signalling

(A) *Left,* schematic of DC (blue) and FRC (grey) in contact in 3D deformable matrix. *Right,* overlay of transmitted light image and immunofluorescence imaging of an FRC and DC in contact. PDPN staining shown in green. Arrow indicates PDPN clustering at cell-cell interface. Scale bar, 20 µm.

(B) Quantitative PCR analysis of CCL19 and CCL21 mRNA in primary FRCs during 7 days of *in vitro* culture.

(C) Quantification of distance migrated in 1 hr time course. Each point represents one DC. *P* value calculated using Mann Whitney U test.

(D) *Left,* schematic showing tracks of WT [red] and *Ccr7*$^{-/-}$ [green] DCs moving on an FRC. *Right,* Stills from time-lapse imaging showing WT [red] and *Ccr7*$^{-/-}$ [green] DCs migrating along FRCs in 3D deformable matrix. Scale bar, 20 μm.

(E) Transmitted light image shows WT DCs interacting with *Icam1*$^{-/-}$ FRCs. Scale bar, 50 μm.

(F) Diagram shows tracks following migration of DCs during 1 hr time course.

(G) Quantification of DC migration along WT and *Icam1*$^{-/-}$ FRC scaffolds in 3D network. Each point represents the path of one DC. *P* value calculated using Mann Whitney U test.

(H) FACS analysis of PDPN surface levels on FRCs and NIH3T3 cells.

(I) FACS analysis of PDPN surface levels on FRCs isolated from LNs of WT and *Pdpn*$^{-/-}$ mice.

(J) FACS analysis of PDPN surface levels on WT FRCs 72 hrs after transfection with either control- or PDPN-targeting siRNA oligos.

Supplemental Movie Legends

Movie S1. Multiphoton Time-Lapse Imaging Shows Migratory DCs Entering the Popliteal LN as Shown in Figure S4B

CFSE-labeled DCs (green), second harmonic (collagen fibres) (red). Frames are 30 s apart and shown at 8 frames/s.

Movie S2. Confocal Time-Lapse Imaging of DC Motility in LN Slices from WT BM → Ub-GFP Mice

The DCs were labeled with cell-tracker dyes; WT DCs are shown in red and *Clec1b*$^{-/-}$ DCs are shown in green. Frames are 90 s apart and shown at 8 frames/s.

Movie S3. Time-Lapse Imaging Shows the Behavior of DCs Cultured in 3D Deformable Matrix and Stimulated with CLEC-2 Ligands

Control DCs are shown first followed by DCs treated with rhodocytin and PDPN-Fc. Frames are 90 s apart and shown at 8 frames/s. The scale bar represents 20 μm.

Movie S4. Time-Lapse Imaging Shows DCs Approaching Branched FRCs in 3D Matrix

Frames are 90 s apart and shown at 8 frames/s.

Movie S5. Time-Lapse Imaging Shows the Migration of WT DCs along WT FRCs Cultured in 3D Matrix

Frames are 2 min apart and shown at 8 frames/s.

Movie S6. Time-Lapse Imaging Shows the Interaction of *Clec1b*$^{-/-}$ DCs with WT FRCs Cultured in 3D Matrix

Frames are 2 min apart and shown at 8 frames/s.

Movie S7. Time-Lapse Imaging Shows the Interaction of WT DCs with *Pdpn*$^{-/-}$ FRCs Cultured in 3D Matrix

Frames are 2 min apart and shown at 8 frames/s.

Supplemental Experimental Procedures

Mice

C57BL/6 mice 6-10 weeks of age were purchased from Jackson Laboratories. *Pdpn*$^{-/-}$ mice on some genetic backgrounds die at birth but some survive on the C57BL/6 background; thus, we used *Pdpn*$^{-/-}$ mice on the C57BL/6 background as described previously (Peters et al., 2012). *Clec1b*$^{-/-}$ mice were generated as previously described (Bertozzi et al., 2010). See supplemental materials and methods for generation of chimeric mice. *Vav1*$^{-/-}$*Vav2*$^{-/-}$*Vav3*$^{-/-}$ mice were generated as previously described (Graham et al., 2009). Animals were maintained under pathogen free conditions in the Animal Facility of the Dana-Farber Cancer Institute in accordance with US National Institutes of Health guidelines.

Generation of fetal liver chimeras

To generate CLEC-2 FLCs, *Clec1b*$^{+}$ and *Clec1b*$^{-/-}$ embryos were collected from females at day E14.5-E18.5 and were processed into a single cell suspension. Recipient B6.SJL-Ptprc^{a}Pep3^{b}/BoyJ mice (Jackson Laboratories, USA) were irradiated at 2x550 Rad, and 10^{6} fetal liver cells were subsequently injected retro-orbitally. The mice received antibiotics (Baytril) for 3 weeks after irradiation and were used in experiments 6-7 weeks after the cell transfer. At this point, total LN DCs were >95% reconstituted by flow cytometric analysis and no *Clec1b*$^{-/-}$ mRNA transcript was dectected in freshly isolated DCs from the *Clec1b*$^{-/-}$ FLCs (data not shown and **Figure 3A**). Furthermore, total LN cellularity and LN DC numbers did not differ significantly between WT and *Clec1b*$^{-/-}$ FLCs (data not shown).

Ubiquitin-GFP (Ub-GFP) BM chimeric mice (WT BM>Ub-GFP host) were generated as follows: BM was prepared from C57BL/6 mice and T, NK, and B cells were depleted via MACS separation using the following biotinylated antibodies: NK1.1 (PK136), CD19 (6D5) and CD3ε (145-2C11) (Biolegend). Recipient Ub-GFP mice (Jackson Laboratories, USA) were irradiated at 2x650 Gy. 24 hrs later, 10^{6} T, B, and NK cell-depleted BM cells were injected i.v. Recipient mice received antibiotic (Baytril) for the first 4 weeks after irradiation and were used for experiments 8 weeks after receiving BM cells.

Endocytosis assays

BMDCs from WT or *Clec1b*$^{-/-}$ FLCs were incubated with 2 μm fluorescent latex beads (Polysciences inc.) in media at 37°C. Beads were added at a concentration of <5 beads per DC. Free beads were washed away and DCs were fixed with 4% PFA, washed with PBS, and bead enumeration was assessed by FACS.

Generation of PDPN-Fc recombinant protein

PDPN extracellular domains were PCR amplified from mouse cDNA using the following primers: F: GTGATATCTGGGAGCGTTTGGTTCTG, R: GGCAGATCTTGGCAAGCCATCTTTC. PCR product was ligated into pFUSE-rFc2 (IL2ss) (InvivoGen) containing a rabbit Fc domain (CH2, CH3, and hinge region) and electroporated into COS-7 cells (ATCC). 24 hrs after electroporation, the culture medium was replaced with complete DMEM (Gibco) supplemented with ultra low Ig-containing serum (Gemini Bio-Products) and 200 mg/ml zeocin for selection (Invivogen). Cells were single cell sorted after 2 weeks of selection. Single cell clones were expanded for large-scale protein production. Supernatant was harvested and purified over a protein G column (GE Healthcare). Proteins were eluted with 0.1 M glycine, pH 2.5, followed by neutralization using 1M Tris, pH 9.0. The proteins were buffer exchanged against PBS.

Transfection of GFP-CLEC-2

Clec1b cDNA was cloned into eGFPC1 (Clontech) from the I.M.A.G.E clone (ATCC, 574821 pT7T3D-Pac). GFP-CLEC-2 fusion was transiently transfected into the human melanoma cell line A375 using Effectene (Qiagen) according to manufacturer’s instructions. Twenty four hrs after transfection, A375 cells were seeded into a 3D deformable matrix at 3:1 ratio with primary FRCs, or plated onto glass-bottomed MatTek dishes for stimulation with CLEC-2 ligand. Single cell clones with high or low expression were generated by single cell sorting transiently transfected A375 GFP-CLEC-2 following 2 weeks of selection with 0.2 mg/ml G418.

siRNA knockdown of PDPN

siRNA complexes were as follows: Con siRNA (all stars control (Qiagen)), PDPN#1 (GCUGCAUCUUUCUGGAUAATT), and PDPN#2 (CGCAGACAACAGAUAAGAATT).

FRCs were transfected with siRNA using oligofectamine (Invitrogen) according to the manufacturers instructions. Efficiency of knockdown was determined by FACS 72 hrs after transfection. PDPN surface expression was measured by FACS.

Analysis of *Clec1b* and *Pdpn* mRNA expression

DCs were sorted from LNs of WT mice by FACS and from WT and *Clec1b*$^{-/-}$ FLCs by MACS using CD11c-biotin beads (Miltenyi, USA). mRNA was extracted from DCs using the RNeasy mini kit (Qiagen) and cDNA template was transcribed using MLV-RT (Invitrogen) according to the manufacturer's instructions. *Clec1b* mRNA expression was determined by qPCR using the following primers: F: AACATCAAGCCCCGGAAACAA, R: GCCACGAGTCCAACAACCA and by RT-PCR using the following primers: F: CAATAGGTCCTGGATGGTCTTC, R: TTACACTGGCTTACCGTGACAG and compared to expression of GADPH or β-actin, respectively. LN stromal cells were sorted as previously described (Fletcher et al., 2011) and PDPN expression levels were determined by microarray (MCBI GEO Database accession number GSE15907).

FITC painting of ear skin

The ears of mice were painted on both sides with 15 μl FITC solution (5 mg/ml Fluorescein isothiocyanate (Sigma) in 1:1 mix dibutylphalate and acetone). Draining cervical LNs were collected after 24 and 72 hrs, digested with collagenase P (Roche 11249002001), dispase (GIBCO 17105-041) and DNase (Roche 10104159001) and analyzed by FACS for the presence of FITC^{+} migratory DCs.

FACS and antibodies

Cells were analyzed using either a FACSCalibur (BD biosciences) or LSR II (BD biosciences). Monoclonal antibodies: α-MHC class II (BD Pharmingen), α-CD40 (Biolegend IC10), α-CD11c (BD Biosciences HL3), α-CD103 (BD Biosciences M290), α-CD326 (Biolegend G8.8), α-CD19 (Biolegend 6D5), α-CD3ε (Biolegend 145-2C11), α-CD8α (Biolegend 53-6.7), αVα2 (Caltag laboratories RM5005), α-CD44 (Biolegend IM7), α-PDPN (hybridoma clone 8.1.1), α-CD45 (Biolegend 30-F11), and α-CD31 (Biolegend MEC13.3). Primary rabbit anti-pMLC (S19) and anti-rabbit-Alexa 555 were purchased from Cell Signaling Technology. Isotype control antibodies were obtained from Biolegend and BD Pharmingen.

Chemotaxis assay

Chemotaxis of BMDCs from WT and *Clec1b*$^{-/-}$ FLCs was measured by migration through a polycarbonate filter of 5 μm pore size in 24-well transwell chambers (Corning Costar). 600 μl media alone as control or containing CCL21 (50 – 500 μM) was added to the lower chamber; 1 x 10^5 DCs were added to the upper chamber and were incubated for 8 hrs at 37°C with 5% CO_2. A 300 μL aliquot of the media from the lower chamber was counted by FACS to assess the percentage of migrating cells.

In vivo and in vitro T cell stimulation assays

For in vivo analysis, WT or *Clec1b*$^{-/-}$ DCs were loaded with 2.5 μg/ml OVA peptide (SIINFEKL) for 2 hrs at 37°C. DCs were then preincubated with rPDPN-Fc or an isotype control antibody. 5x10^5 DCs were injected into the footpad in 10 ml PBS containing 10 ng/ml LPS. OT-1 splenocytes were labeled with CFSE by resuspending cells in RPMI at 10x10^6 cells/ml plus 2.5 μM CFSE, incubated at 37C for 10 min, washed three times,

and then counted. 24 hrs after DC injection, the mice were intravenously injected with 1.5×10^6 CFSE-labeled OT-1 T cells in 200 ml PBS. Axillary (distal) and popliteal (draining) LNs were removed 48 hrs later and T cell proliferation was evaluated by measuring CFSE dilution among OT-1 T cells (identified by TCR clonotype) using FACS. Division index was calculated as the average number of divisions among cells that had divided at least one time. For in vitro analysis, a single cell suspension of red blood cell-depleted splenocytes was prepared from OT-1xRag1$^{-/-}$ mice. The OT-1 splenocytes were then labeled with CFSE by resuspending cells in RPMI at 10×10^6 cells/ml plus 2.5 μM CFSE and incubating at 37°C for 10 min. Cells were washed twice in cold RPMI containing 10% FCS and counted. BMDCs from WT and *Clec1b*$^{-/-}$ FLCs were loaded with 2.5 μg/ml OVA peptide (SIINFEKL, AnaSpec) and then co-cultured with CFSE-labeled OT-1 T cells in culture media (RPMI; 10% FCS, 1% penicillin/streptomycin) for 48 hrs. T cell proliferation was assessed by measuring CFSE dilution among OT-1 T cells (identified by TCR clonotype) by FACS. Division index was calculated as above.

Analysis of RhoA and Rac1 activation

A375 single cell clones expressing either low or high levels of GFP-CLEC-2 were treated with CLEC-2 ligands (Rhodocytin, 200 nM and PDPN-Fc, 0.4 μg/ml) for 5 min then washed with PBS and lysed on ice. Cell lysates were incubated for 5 hrs with GST-Rhotekin-RBD or GST-PAK1-PBD bound to glutathione agarose. Beads were then extensively washed and bound proteins were eluted by boiling the beads in LDS sample buffer. Levels of RhoA-GTP and Rac1-GTP were evaluated by western blot and compared to total RhoA and total Rac-1 in whole cell lysates.

Analysis of pMLC

3D matrices seeded with DCs and FRCs or DCs alone were fixed after 18 hrs with 4% PFA for 2 hrs at 4°C, permeabilized with 0.25% Triton in PBS for 30 min at room temperature, then blocked with 3% BSA 0.01% Tween-20 in PBS for 2 hrs at room temperature. Primary rabbit anti-pMLC (S19) (Cell Signaling Technology) was added in blocking buffer at 1:100 dilution for 3 hrs at room temperature. Cultures were washed 3x in blocking buffer for 10 min each. Anti-rabbit Alexa 555 was then diluted 1:300 in blocking buffer and incubated for 2 hrs at room temperature. CD11c-FITC conjugated Ab was used at 1:50 to visualize DCs. Images were taken using Zeiss LSM 510 confocal microscope. ImageJ software was utilized to quantify the intensity of pMLC staining in DCs.

Immunofluorescence staining of tissues and cells

For staining of the 3D deformable matrices, cultures were washed with PBS then fixed with 4% PFA for 1 hr, permeabilized with 0.2% TRITON in PBS for 20 minutes and blocked with 3% BSA for 2 hrs before staining. PDPN was visualized using clone 8.1.1 conjugated to Alexa 647 (Biolegend). F-actin and cell nuclei were visualized using TRITC-phalloidin and DAPI, respectively (Invitrogen). For whole mount staining of ear skin, ears were split and ventral halves were used for staining. Ears were fixed in 1% PFA for 3 hrs, permeabilized with 0.1% TRITON for 30 min and blocked in 1% BSA for 3 hrs before staining. Monoclonal antibodies (8.1.1 conjugated to Alexa 647) and Lyve-1 (eBioscience FITC-conjugated) were used. F-actin was visualized with TRITC-phalloidin.

Imaging

Confocal imaging was conducted using a Zeiss LSM510 Meta microscope with the 20x objective. Time-lapse microscopy was also conducted using a Zeiss LSM510 Meta microscope with the transmitted light detector. MP-IVM was conducted as previously described (Gonzalez et al., 2010). BMDCs were treated with 10 ng/ml LPS for 18 hrs, labeled with 5 μM CFSE (Invitrogen), and then injected into recipient mice. Mice received 2.5×10^5 labeled DCs injected into the footpad in 10 μl PBS 18 hrs before MP-IVM imaging was conducted.
